# Comparison of Early Changes in Ocular Surface and Inflammatory Mediators between Femtosecond Lenticule Extraction and Small-Incision Lenticule Extraction

**DOI:** 10.1371/journal.pone.0149503

**Published:** 2016-03-03

**Authors:** Chi Zhang, Hui Ding, Miao He, Lina Liu, Liangping Liu, Gang Li, Bing Niu, Xingwu Zhong

**Affiliations:** 1 Zhongshan Ophthalmic Center and State Key Laboratory of Ophthalmology, Sun Yat-sen University, Guangzhou 510060, China; 2 Department of Ophthalmology, The First People’s Hospital of Foshan, Foshan 528000, China; 3 Hainan Eye Hospital, Zhongshan Ophthalmic Center, Sun Yat-sen University, Haikou 570311, China; 4 Shanghai Key Laboratory of Bio-energy Crops, School of Life Science, Shanghai University, Shanghai 200444, China; 5 Schepens Eye Research Institute, Massachusetts Eye and Ear Infirmary, Department of Ophthalmology, Harvard Medical School, Boston, MA 02114, United States of America; The Chinese University of Hong Kong, HONG KONG

## Abstract

**Purpose:**

To evaluate the short-term changes in ocular surface measures and tear inflammatory mediators after femtosecond lenticule extraction (FLEx) and small-incision lenticule extraction (SMILE) procedures.

**Methods:**

Eighteen subjects (18 eyes) underwent FLEx and 23 subjects (23 eyes) underwent SMILE in this single-center and prospective study. Central corneal sensitivity, Schirmer I test (SIT), noninvasive tear breakup time (NI-TBUT), tear meniscus height, corneal fluorescein (FL) staining, and ocular surface disease index (OSDI) were assessed in all patients. Concentrations of interleukin-1α (IL-1α), tumor necrosis factor-α (TNF-α), nerve growth factor (NGF), interferon-γ (IFN-γ), transforming growth factor-β1 (TGF-β1) and matrix metalloproteinase-9 (MMP-9) in collected tears were measured by multiplex antibody microarray.

**Results:**

Central corneal sensitivity was reduced in both groups, but the scores in the SMILE group were higher than those in the FLEx group at all time points postoperatively (P<0.01). Lower FL scores and longer NI-BUT were observed in the SMILE group 1 week after surgery (P<0.05). OSDI scores in both groups increased rapidly at 1 day and 1 week postoperatively, then returned to their preoperative levels within 1 month (P<0.05). There were no significant differences in SIT or tear meniscus height between the two groups. Lower and faster recovery of tear NGF, TGF-β1 and IL-1α concentration were found in the SMILE group compared to the FLEx group postoperatively. No significant difference was found in tear TNF-α, IFN-γ and MMP-9 for either group before or after surgery. Tear NGF, TGF-β1 and IL-1α show a correlation with ocular surface changes after FLEx or SMILE surgery.

**Conclusion:**

SMILE has superiority over FLEx in early ocular surface changes and NGF, TGF-β1 and IL-1α may contribute to the process of ocular surface recovery.

**Trial Registration:**

ClinicalTrials.gov NCT02540785

## Introduction

The use of femtosecond (FS) laser has become one of the most significant technological advancements in refractive surgery. A breakthrough FS laser-assisted myopic and myopic astigmatic correction procedure can now be performed using a prototype of the VisuMax femtosecond system. This first all-in-one FS-laser system was designed to perform the refractive lenticule extraction (ReLEx) procedures, femtosecond lenticule extraction (FLEx) and small-incision lenticule extraction (SMILE). In FLEx, a corneal flap is created by the FS laser (similar to LASIK) and lifted, allowing lenticule removal [1–3]. For SMILE, a truly flapless procedure, only a small—2–4mm—incision is made, through which the lenticule is removed [4–8].

Ocular surface disruption during corneal refractive surgery is commonly considered to be closely related to the development of dry eye. Multiple etiologies contribute to this ocular surface disruption, including the flap creation and stromal ablation involved in previous refractive surgery techniques [9]. Corneal nerve damage has been considered the main cause of dry eye, due to disrupted afferent sensory nerves, reduced blink reflex, and increased tear evaporation leading to tear film instability [10–13]. In addition, postoperative inflammatory mediator fluctuations are also a key factor related to ocular surface damage. Extensive research has described the effects of cytokines, chemokines and growth factors in modulating corneal wound healing, cell migration, and apoptosis on the ocular surface after refractive surgery [14–18].

For both FLEx and SMILE, stromal ablation has been replaced by refractive lenticule removal. In terms of corneal flap formation, FLEx still requires an epithelial-stromal flap, while SMILE employs only a small incision to extract the lenticule. Hence, we hypothesize that SMILE will have less effect on patients’ ocular surface markers and inflammatory mediators, compared to FLEx. In support of this hypothesis, previous studies have reported that more damage to the sub-basal nerve plexus of the cornea and more changes in ocular surface evaluations were found after FLEx than after SMILE [5, 19–21]. In this study, we have focused on postoperative changes to tear inflammatory mediators and the relationship of FLEx and SMILE to dry eye.

To test our hypothesis, we conducted a prospective clinical study in patients who underwent FLEx or SMILE. Ocular surface parameters and inflammatory mediators were assessed and compared between the different types of surgery.

## Materials and Methods

### Study Oversight

The study was reviewed and approved by the Ethics Committee of Hainan Eye Hospital, Zhongshan Ophthalmic Center of Sun Yat-sen University in compliance with the tenets of the Declaration of Helsinki in January 2014 (protocol number 2014–005) (S1 and S2 Protocols). We thought only the permission of the ethics committee was sufficient. Therefore, we didn't register before until we are required to register after submission. The study was registered at www.clinicaltrials.gov (registration number NCT02540785). The authors confirm that all ongoing and related trials for this intervention are registered. All patients provided written consent to participate in the study and finished all postoperative follow-up visits.

### Study Design

This is a prospective, non-randomized, controlled, single-center study conducted at Hainan Eye Hospital, Zhongshan Ophthalmic Center of Sun Yat-sen University. Subjects were enrolled between April 2014 and December 2014 and were followed for one month (S1 TREND Checklist).

#### Subjects

The recruited patients who underwent bilateral SMILE (n = 23) or FLEx (n = 18) surgeries to correct myopia or myopic astigmatism. The enrollment criteria were: minimum age of 18 years (range from 18 year to 25 years); corneal thickness more than 500 μm and calculated residual stromal bed after treatment greater than 300 μm; preoperative spherical equivalent refraction between -2.00 diopter (D) and -6.50 D; preoperative cylindrical equivalent refraction between -0.25 D and -1.50 D; preoperative corneal curvature from 41.0 D to 46.0 D with a regular topographic pattern; monocular best corrected visual acuity of 20/20 or better and stable refractive error (less than 0.5 D change) for 24 months before surgery. Exclusion criteria were as follows: systemic disease that contraindicated the surgery (such as diabetes, glaucoma and systemic collagen vascular disease); corneal abnormality or disease; a history of tear supplement usage or contact lens wear during the past year. All surgeries were performed by the same experienced surgeon (Xingwu Zhong) following standard procedures under topical anesthesia. Data from one eye of each subject were included in the statistical analysis, to prevent bias. A random number table was used to determine which eye of each patient was included.

#### Preoperative Examination

Preoperative assessment included general medical and ophthalmic histories, current medications, and assessment of uncorrected distance visual acuity (UDVA), corrected distance visual acuity (CDVA), intraocular pressure (IOP), corneal topography (HUMPHREY HCT993, Orbscan; Bausch & Lomb Inc, Rochester, NY, USA), keratography (TOPCON OM-4, Japan), axial length (Ocuscan Rxp, Carl Zeiss Meditec AG), dilated fundoscopy and slit-lamp microscopy. All visual acuity measurements were performed using Snellen charts.

#### ReLEx FLEx procedure

The VisuMax femtosecond laser system (Carl Zeiss Meditec AG, Jena, Germany) with a 500 kHz repetition rate was used to perform FLEx surgery. Four femtosecond incisions were created in succession: the posterior surface of the refractive lenticule (spiral in), the lenticule border, the anterior surface of the refractive lenticule (spiral out), and the corneal flap in the superior region. After the suction was released, the flap was opened using a thin, blunt spatula and the free refractive lenticule was subsequently grasped with a forceps and extracted, after which the flap was repositioned carefully.

The planned flap thickness with superior hinge and 50 degrees in cordal length was 120 μm. The flap diameter was 7.5 μm and the lenticule diameter 6.5 mm. The optical zone size was 6.5 μm. The spot spacing and tracking spacing were 4.5 μm for the lenticule and 2.0 μm for the lenticule side cut. The energy of the femtosecond laser was 140 nJ.

#### ReLEx SMILE procedure

ReLEx SMILE surgery was also performed using the VisuMax femtosecond laser system with a 500 kHz repetition rate, as described by Gao [22]. The only difference from the ReLEx FLEx procedure was to make a small incision in the last step, instead of creating a corneal flap. Once the four incisions were created, suction was released automatically and the anterior and posterior refractive surfaces were separated by a thin, blunt spatula. The refractive lenticule was extracted through the incision using forceps.

The optical zone size was 6.5 mm. The anterior lenticule surface was 120 μm deep. The small incision was located in the 120° position, with 50 μm cordal length (the side-cut incision with a circumferential length of 4.0–5.0 mm and angle of 90°). The spot spacing and tracking spacing were 4.5 μm for the lenticule, 2.0 μm for the lenticule side cut, 3.0 μm for the small incision and 2.0 μm for the small incision side cut. The energy of the femtosecond laser was 140nJ.

Standard postoperative treatment consisted of 0.3% tobramycin/dexamethasone (TobraDex, Alcon) eyedrops, 0.5% levofloxacin (Cravit, Santen) eyedrops and sodium hyaluronate (HYCOSAN,URSAPHARM Arzneimittel GmbH) four times a day for one week. Tobradex and Cravit were suspended after one week, but artificial tear eyedrops were applied as required until one month.

#### Ocular Surface Measurement

Ocular surface parameters evaluated included Schirmer I test (SIT) without anesthesia, corneal fluorescein (FL) staining, noninvasive tear breakup time (NI-TBUT), tear meniscus height, ocular surface disease index (OSDI) and central corneal sensitivity. All assessments were performed prior to the surgery and 1 day, 1 week, and 1 month postoperatively. NI-TBUT and tear meniscus height were assessed by the Keratograph 5 (Oculus, Wetzlar, Germany). The Cochet-Bonnet corneal esthesiometer (Luneau Ophthalmologie Chartres, Cedex, France) was used to assess central corneal sensitivity. FL staining and central corneal sensitivity were not performed 1 day after the surgery because of the potential for corneal damage. All ocular surface measurements were performed by the same doctor but not in a masked manner because it is not difficult to figure out what kind of surgery the patients underwent by slit lamp.

#### Tear Collection

A nonstimulated tear sample was collected by using disposable 5-mL microcapillaries (Microcaps 5 mL; Drummond Scientific, Broomall, PA). Tear collection was performed from the inferior marginal region without irritation of the cornea, conjunctiva or lid margin. A 20μl sample was obtained, transferred to a 0.5 ml microtube, and stored at -80°C until processing.

#### Inflammatory Mediators Assay

Interleukin-1α (IL-1α), tumor necrosis factor- α (TNF-α), nerve growth factor (NGF), interferon-γ (IFN-γ), transforming growth factor-β1 (TGF-β1) and matrix metalloproteinase-9 (MMP-9) in collected tears were measured by a Quantibody Human Inflammation Array I kit (RayBiotech, Inc. Norcross, GA) according to the manufacturer’s instructions. In brief, antibodies against the inflammatory mediators were spotted onto the cytokine array. After incubation with tear samples for 2 hours, biotin-conjugated secondary antibodies were added for 1 hour. Then the Cy3 dye-conjugated streptavidin was added for another 1 hour. The signals were captured by GenePix 4000B (Bio-Rad Laboratories, Hercules, CA) and analyzed by Quantibody® Q-Analyzer software (RayBiotech, Inc. Norcross, GA).The concentration was quantified according to the standard curves generated from standards provided by the manufacturer.

#### Statistical Analysis

Data were analyzed using SPSS 19.0 software (SPSS, Chicago, IL, USA). Compared with preoperative level, a ANOVA for repeated measurements was applied to analyze the data in each group. Comparisons between the two groups were performed by Independent samples t-test (normally distributed data) or Mann-Whitney U test (non-normally distributed data). The adjusted level was established by Bonferroni test. Pearson or Spearman rank correlation was used to analyze the correlations between tear inflammatory mediators and ocular surface parameters. *P* value of less than 0.05 was considered statistically significant. Data are presented as mean ± standard deviation (SD).

## Results

**Fig 1:**
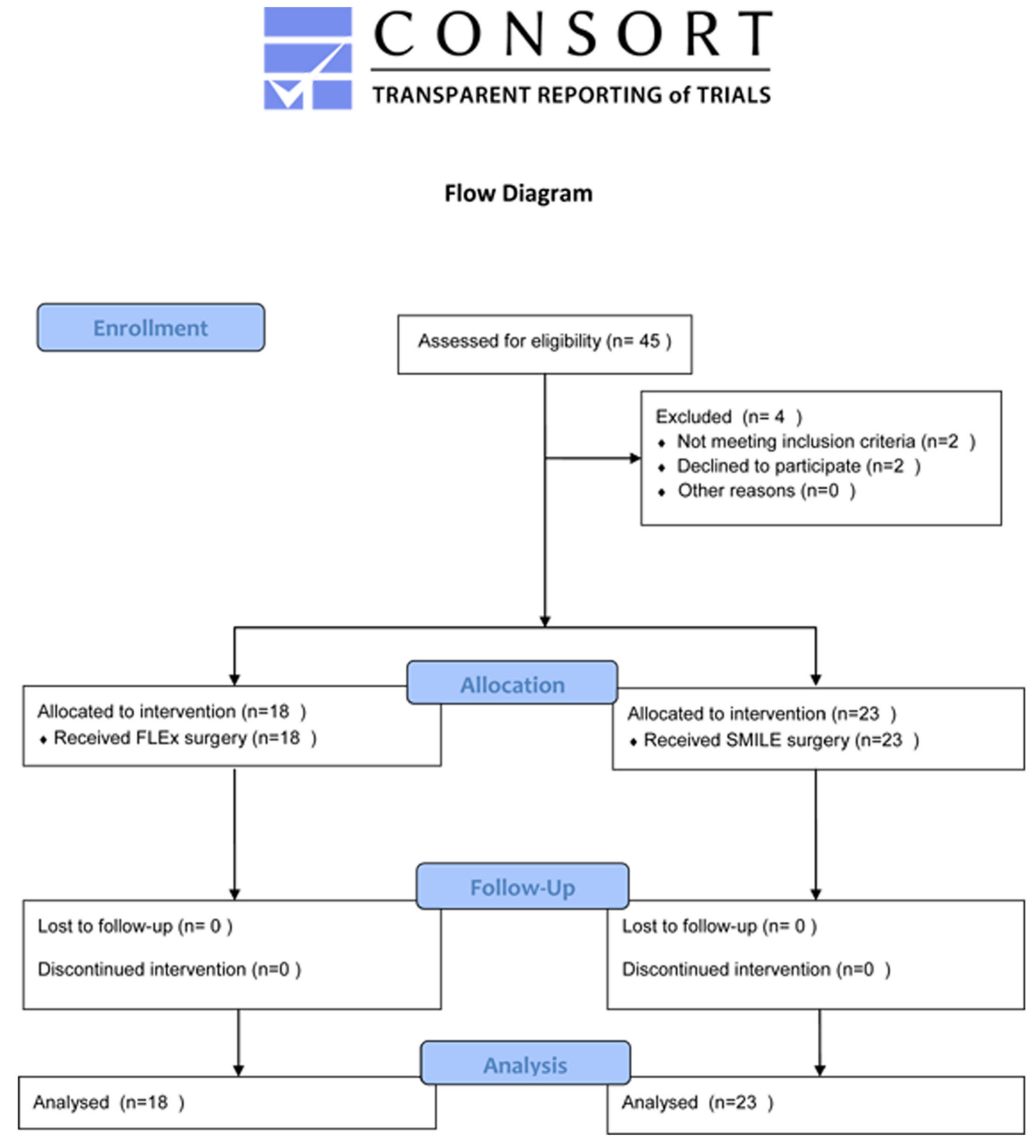
Flow diagram of patient recruitment.

There were 41 patients enrolled in the study (Fig 1). All subjects were matched for age, initial uncorrected visual acuity, best spectacle-corrected visual acuity, IOP, spherical equivalent, central corneal thickness, and corneal curvature. The preoperative and 1 month postoperative clinical characteristics, visual and refractive outcomes of all patients in both groups are shown in Table 1, which were not significantly different.

**Table 1:** Preoperative and 1 month postoperative characteristics of the study participants.

Parameter	FLEx	SMILE	P-Value
Patients (n)	18	23	
Eyes (n)	18	23	
Male/Female	9/9	13/10	
Age (years)	20.61±3.74	20.26±3.09	0.769
Preoperative			
IOP (mmHg)	14.89±1.45	14.09±1.56	0.693
CCT (μm)	559.05±24.42	551.05±26.47	0.645
K value	42.84±1.27	43.63±1.71	0.367
SE (D)	−4.31±1.85	−4.64±1.64	0.732
UDVA (logMAR)	0.98±0.34	0.92±0.29	0.534
CDVA (logMAR)	−0.10±0.04	−0.08±0.05	0.067
1 month postoperative			
IOP (mmHg)	10.78±1.36*	10.21±1.23*	0.526
CCT (μm)	490.15±17.72*	485.18±18.33*	0.445
K value	39.95±1.22*	40.33±1.31*	0.475
SE (D)	−0.33±0.61**	−0.29±0.47**	0.125
UDVA (logMAR)	−0.07±0.06**	−0.06±0.04**	0.091
CDVA (logMAR)	−0.09±0.03**	−0.09±0.04**	0.088

IOP: intraocular pressure; CCT: central corneal thickness; SE: spherical equivalent; UDVA: uncorrected distance visual acuity; CDVA: corrected distance visual acuity. logMAR: logarithm of mininal angle resolution. All data except patients, eyes and gender are presented as mean ± standard deviation (SD) of all eyes in relevant groups.

*P<0.05, significant differences compared with preoperative level.

**P<0.01, significant differences compared with preoperative level.

### Ocular Surface Assessment

#### Corneal sensitivity

**Fig 2:**
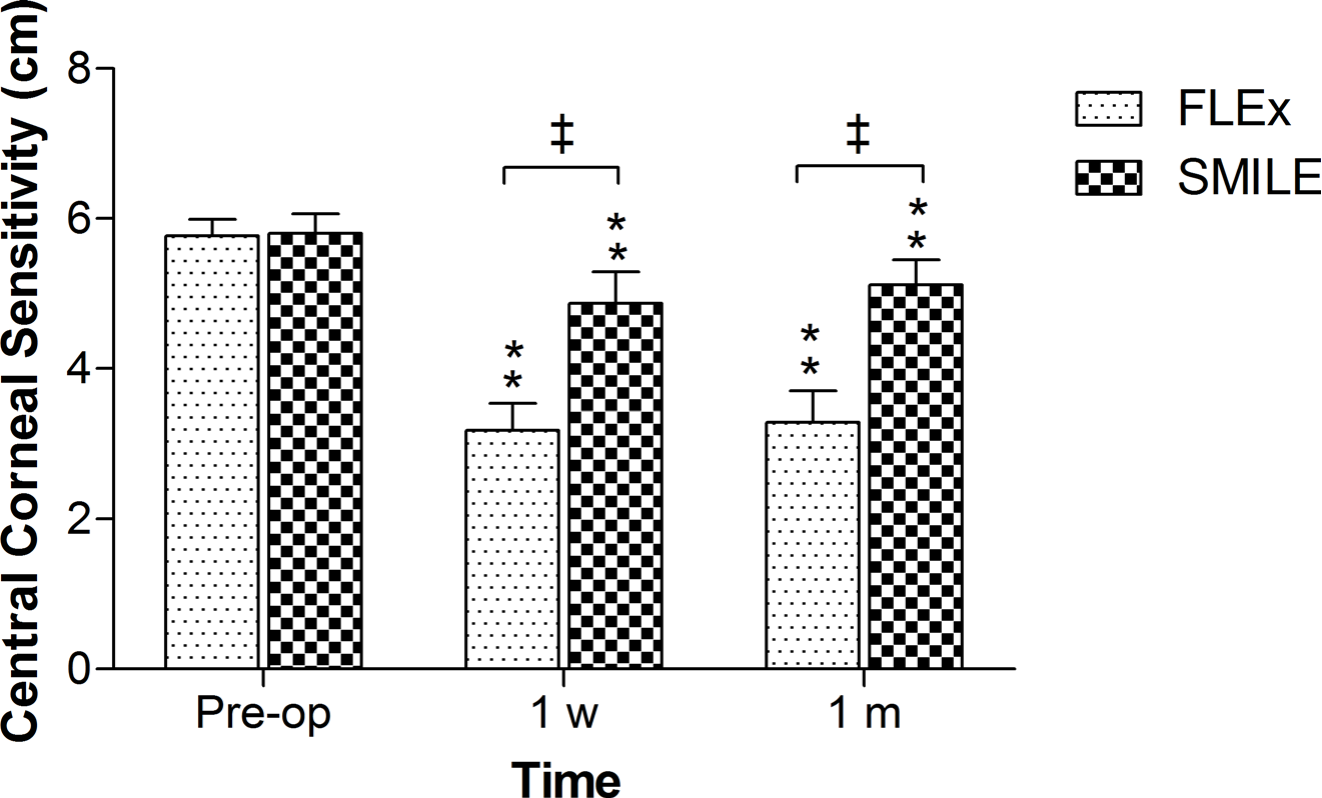
Time dependent changes in the central corneal sensitivity after FLEx and SMILE. *P<0.05, significant differences compared with preoperative level. **P<0.01, significant differences compared with preoperative level. ‡ P<0.01, significant differences between groups.

In both FLEx and SMILE groups, central corneal sensation was significantly decreased at 1 week and 1 month postoperatively, compared with preoperative levels (P<0.01). Moreover, there was a significantly greater loss of central corneal sensitivity in the FLEx group than in the SMILE group at 1 week and 1 month after surgery (P<0.01). (Fig 2).

#### Tear film evaluation

**Fig 3:**
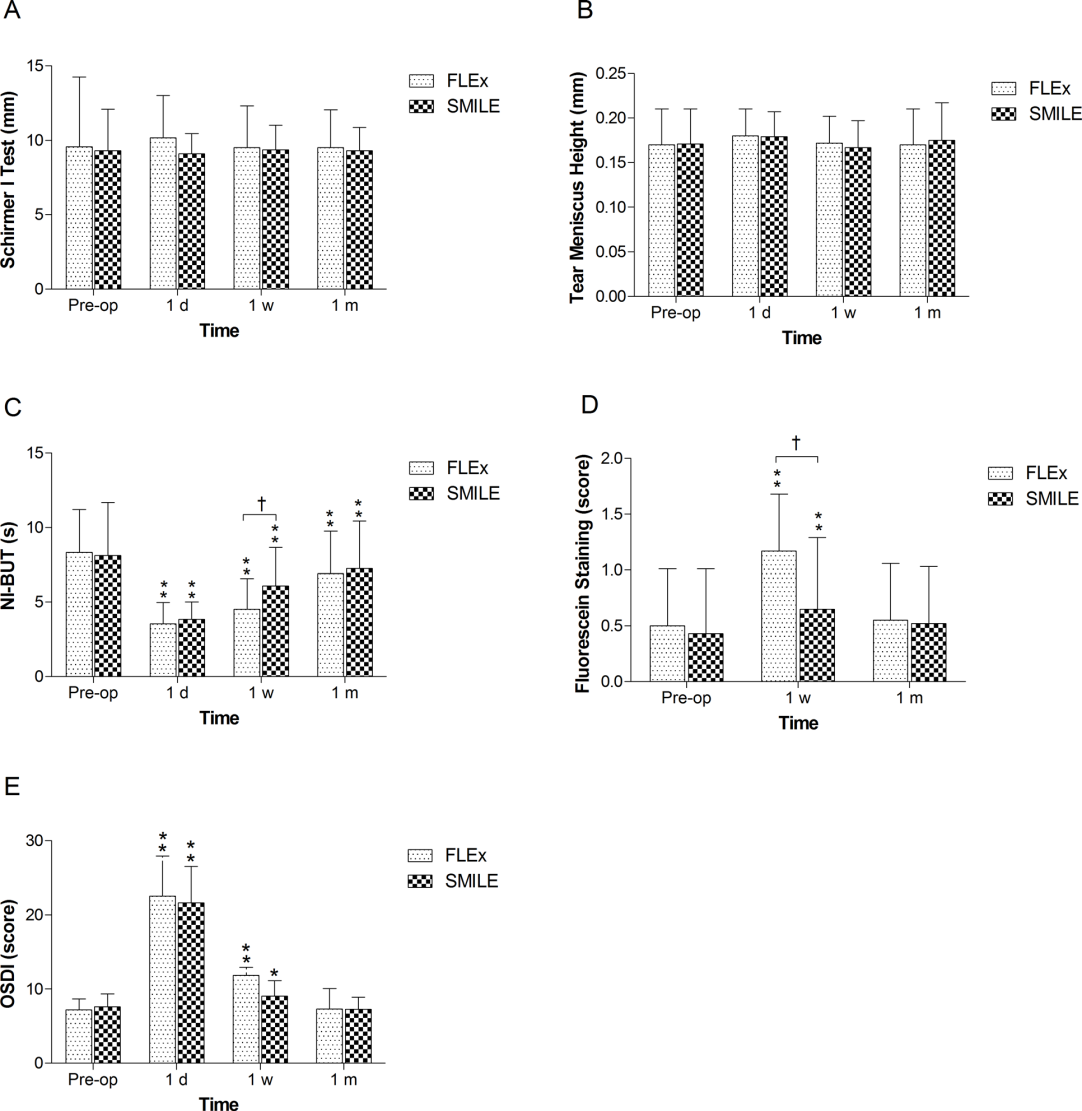
Time dependent changes in the tear film parameters after FLEx and SMILE. *P<0.05, significant differences compared with preoperative level. **P<0.01, significant differences compared with preoperative level. † P<0.05, significant differences between groups

There was no difference in preoperative tear film parameters including Schirmer I test, tear meniscus height, NI-TBUT and OSDI score between FLEx and SMILE eyes (Fig 3).

For the Schirmer I test and tear meniscus height, no significant difference was found between any time points in either group, or between the two groups (Fig 3A and 3B).

The NI-TBUT in both groups was remarkably attenuated postoperatively compared with the preoperative measurement. The lowest value was 3.53±1.44 seconds in the FLEx group and 3.84±1.17 in the SMILE group at 1 postoperative day. At 1 week after surgery, the NI-TBUT increased to 6.07±2.58 seconds in the SMILE group, compared to 4.52±2.05 seconds in the FLEx group (P<0.05). However, there was no significant difference in NI-TBUT between the two treatment groups at 1 month after surgery. On the contrary, corneal staining was found to be increased at 1 week after surgery (P< 0.01) and recovered to almost the baseline level in both groups at 1 month postoperatively. Compared with SMILE, corneal staining was more severe in the FLEx group at 1 week after surgery (P< 0.05). (Fig 3C and 3D).

We found that OSDI scores decreased significantly at 1 day and 1 week postoperatively, compared with the baseline value in both the FLEx and SMILE groups (P<0.01). The highest score was at 1 postoperative day, which was consistent with the long NI-TBUT. No significant differences were found between the two groups. (Fig 3E)

### Tear Inflammatory Mediators

**Fig 4:**
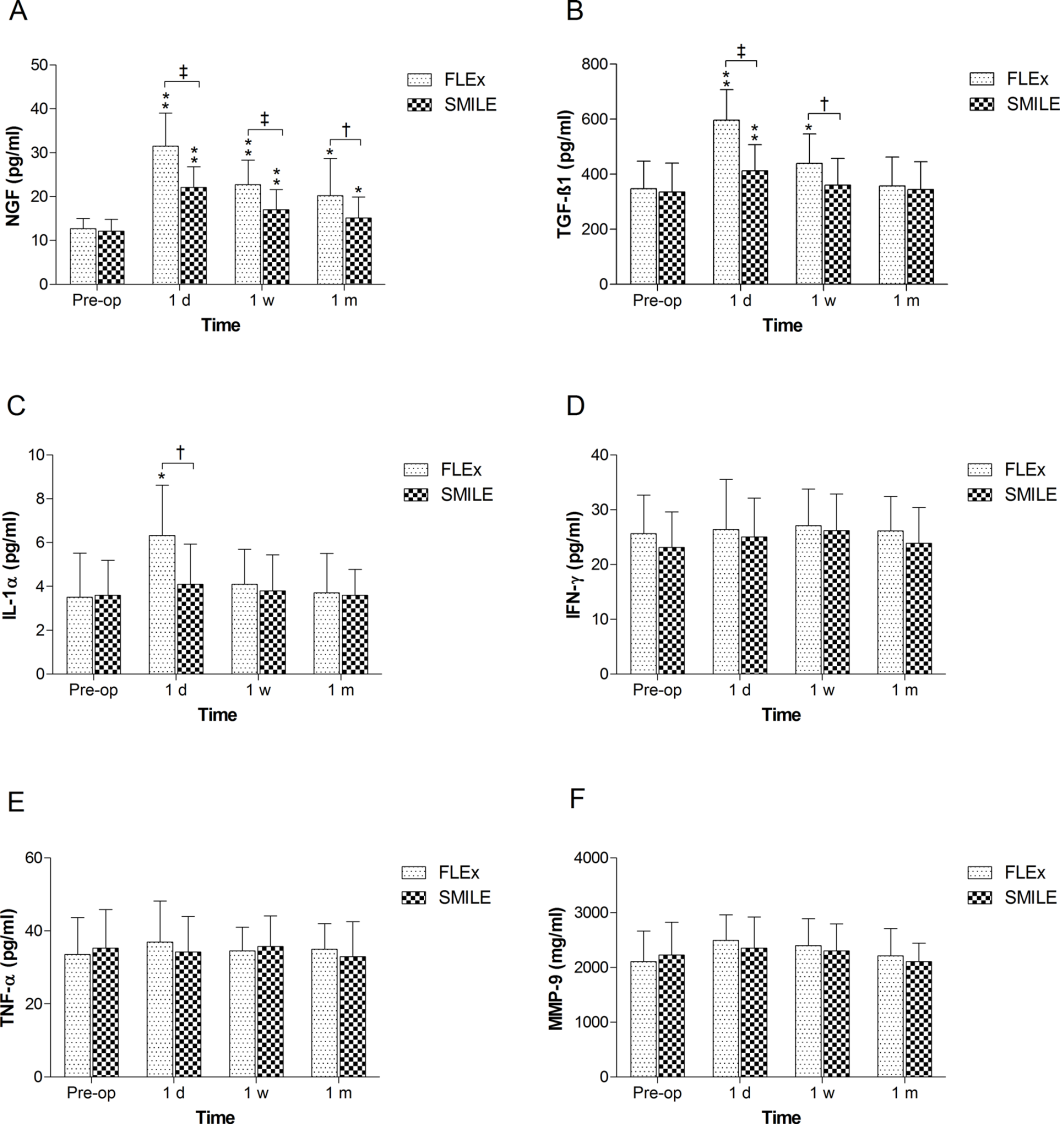
Time dependent changes in concentrations of inflammatory mediators in tear after FLEx and SMILE. *P<0.05, significant differences compared with preoperative level. **P<0.01, significant differences compared with preoperative level. † P<0.05, significant differences between groups. ‡ P<0.01, significant differences between groups.

Our study found no significant difference in the tear inflammatory mediators IL-1, TNF-α, NGF, IFN, TGF-β1 or MMP-9 between the FLEx and SMILE groups prior to surgery (Fig 4).

The fluctuations in tear NGF concentrations in the FLEx and SMILE groups were similar to NI-TBUT. In both groups, NGF was increased significantly throughout the follow-up period, compared with the preoperative levels (P<0.01). The peak values of 31.5±7.57 pg/ml (FLEx) and 22.1±4.69 pg/ml (SMILE) were measured 1 day after surgery. There was also a significant difference between the level of NGF in the FLEx and SMILE groups at all of the postoperative time points (P<0.05, Fig 4A).

Tear TGF-β1 was significantly elevated in the FLEx group at the 1 day and 1 week postoperative assessments and in the SMILE group 1 day after surgery (P<0.01). The greatest concentration of TGF-β1 in both the FLEx group (595.41±111.72 pg/ml) and SMILE group (412.32±95.58 pg/ml) was observed at 1 day postoperatively. While the TGF-β1 level in the SMILE group declined significantly at 1 week postoperatively, it remained elevated until 1 month after surgery in the FLEx group. Moreover, the level of TGF-β1 was significantly higher in the FLEx group at 1 day and 1 week after surgery (P<0.01, Fig 4B).

The concentration of IL-1α increased in the FLEx group at 1 day postoperatively compared with the baseline level (P< 0.05) and the SMILE group (P< 0.05, Fig 4C). There was no significant change in the SMILE group at any time point. Moreover, no significant difference was found in the levels of tear TNF-α, IFN-γ or MMP-9, either between the two groups or before and after surgery (Fig 4D, 4E and 4F).

### Correlation Between Inflammatory Mediators and Ocular Surface Changes

Correlation analysis was performed between inflammatory mediators and the ocular surface parameters. NGF, TGF-β1, and IL-1α were found to be significantly correlated with OSDI, NI-BUT or FL, but there were no significant correlations between TNF-α, IFN-γ and MMP-9 and any of the ocular surface measures (Table 2).

**Table 2:** Correlations coefficients of inflammatory mediators and some ocular surface parameters.

	FLEx						SMILE			
	OSDI		NI-BUT		FL		OSDI		NI-BUT	
	*r*	P	*r*	P	*r*	P	*r*	P	*r*	P
NGF	0.491	0.02	-0.4	0.003			0.593	0.001	-0.352	0.003
TGF-β1	0.563	0.001			0.361	0.03			-0.307	0.01
IL-1α	0.459	0.001								

### Complications

No serious complications, such as flap tears, corneal ectasia, buttonholes, or incomplete passes, were observed during this study. No eyes experienced delayed visual recovery or visual loss, and no flap folds or haze were seen during follow-up.

## Discussion

In the current study, we observed that central corneal sensitivity, NI-BUT, corneal fluorescein staining, tear NGF, TGF-β1 and IL-1α showed significant differences between the FLEx and SMILE groups after surgery. Postoperative OSDI scores decreased compared to baseline in both treatment groups. The greatest differences from baseline were observed 1 day after surgery. In support of our hypothesis, these changes were less severe in the SMILE group.

Corneal nerves enter the cornea through the peripheral mid-stroma, then branch into smaller fibers. The stromal nerve fibers penetrate Bowman’s layer, proceed parallel to the superficial cornea between the basal epithelium and Bowman’s layer, and ultimately terminate in the corneal epithelium [23, 24]. Severing corneal nerves prevents transmission by sensory receptors and leads to a decrease in corneal sensation [25]. The significant potential advantage of SMILE surgery is the "flapless" aspect, in which only a small (about 50°) side-cut incision is made, and no lifting occurs, as compared to a 330° side-cut flap, with lifting and repositioning, in the FLEx procedure. A small incision means less corneal nerve damage, which results in milder effects on corneal sensation [26, 27]. In our present study, we found that sensitivity in the central corneal region was greater in the SMILE group than in the FLEx group at all the follow-up time points. This is consistent with the previous findings. For example, Wei *et al*. reported that postoperative corneal sensation after SMILE was better than after FLEx at 1week, 1 month and 3 months after surgery [5]. Vestergaard *et al*. observed that sub-basal nerve density and corneal sensitivity were more attenuated 6 months after FLEx than SMILE [20]. Ishii *et al*. also showed a similar trend in their comparison of the two surgeries with follow-up times of up to 1 year [28]. Wei *et al*. stated that central and peripheral corneal sensitivity after FLEx and femtosecond laser-assisted laser in situ keratomileusis (FS-LASIK)—another flap-required surgery—were similar [5]. Their data indicate that flap creation and displacement contribute to the damage of corneal nerves, regardless of the subsequent procedure [29].

Dry eye has become the most prevalent sequela and a major cause of patient dissatisfaction after corneal surgery [30, 31]. The transection of corneal nerves is a key factor in the multifactorial pathophysiology of dry eye, and the extent of the damage is generally correlated with severity. The decrease in corneal innervation reduces reflex-induced lacrimal secretion and blinking rate, which results in attenuated tear production and increased evaporative loss, leading to tear film instability and dry eye [32, 33]. In our evaluation of the tear film, Schirmer I test, FL, and NI-BUT returned to the baseline level more quickly in the SMILE group than in the FLEx group, which suggests that deterioration of corneal sensation was less severe after SMILE than after FLEx. However, OSDI score and tear meniscus height in the SMILE group were not significantly different from the FLEx group. Ishii *et al*. also stated that dry eye symptoms and signs were no less severe in SMILE-treated eyes than in FLEx-treated patients [28]. One possible explanation for this unexpected similarity may be that the subjective symptoms and objective assessment for dry eye do not always exhibit meaningful correlations [34, 35].

Refractive surgeries usually result in the release of multiple inflammatory mediators, which play a significant role in corneal wound healing and tear film integrity [13, 36, 37]. To our knowledge, there have been no other reports comparing the inflammatory responses after FLEx and SMILE surgeries. In this study, we found that NGF, TGF-β1 and IL-1α changed significantly after either FLEx or SMILE surgery.

Our previous study and other reports are consistent with the current finding, in that NGF levels increased after PRK [38], Epi-LASEK [39], LASIK [38], FS-LASIK [22] and SMILE [22]. NGF is a neurotrophin involved in the differentiation and survival of peripheral nerve tissue. NGF has been identified in human corneal epithelial and stromal tissue both *in vivo* and *in vitro* [40, 41], which is also believed to be responsible for maintenance of normal corneal sensitivity, integrity of the corneal epithelium, and acceleration of corneal wound healing [42, 43]. Cells of the epithelium and keratocytes are capable of producing and storing NGF, which is released after injury to the corneal epithelium and stroma [42]. Proinflammatory cytokines activated in response to corneal damage also can amplify the expression of NGF through signaling cascades [44]. The damage to the corneal epithelium and stroma that occurs when using FS to create an incision and lenticule in both FLEx and SMILE, resulted in the over expression of NGF compared with baseline levels. In addition, our data indicate that the NGF level was higher in the FLEx group than in the SMILE group at all of the follow-up time points. During both FLEx and SMILE, the epithelium, Bowman's layer and the anterior stroma are inevitably injured because of the creation of the lenticule and a 330° corneal flap (FLEx) or a 50° incision (SMILE). The more extensive disruption of the cornea in FLEx than in SMILE may result in a more robust inflammatory response and elevated NGF concentration. Evidence from previous study indicates similar effects. Lee *et al*. reported that NGF levels were higher in PRK than in LASIK subjects [38], which may resulted from the more severe damage for anterior cornea in PRK compared to LASIK. Moreover, the correlations between NGF and OSDI and NI-BUT also demonstrated that the postoperative NGF increase in the FLEx group might result in more severe ocular surface damage. The lower NGF levels in early post-SMILE eyes compared with post-FLEx eyes may imply that SMILE is less detrimental and invasive than FLEx. However, the exact mechanism for this difference need to be further studied.

In this study, a postoperative elevation of tear TGF-β1 was observed in both FLEx and SMILE patients, but the expression was higher and slower to recover after FLEx surgery. The correlation analysis indicates that increased TGF-β1 is related to the more serious disruption of the ocular surface in FLEx than SMILE. TGF-β1 is a multifunctional cytokine which plays a vital role in several components of corneal wound healing after refractive surgery, including keratocyte activation, myofibroblast transformation, fibronectin synthesis, and collagen gel contraction [45–48]. TGF-β1 is mainly localized to the intact corneal epithelium, but is also expressed in Bowman's layer and the stroma during wound healing [49, 50]. In addition, TGF-β1 mRNA and protein have been detected in human lacrimal gland [51]. In our study, the larger area of the cornea destroyed by the epithelial-stromal flap creation in FLEx may activate a stronger inflammatory response and greater release of TGF-β1 from cornea, compared with the small incision necessary for SMILE. In addition, NGF has been reported to be involved in modulating inflammation and enhancing the release of TGF-β1 [41, 52, 53], which is consistent with the dynamic changes in NGF level in both surgery groups. Moreover, we speculate that the relatively severe injury to the cornea in FLEx, compared with SMILE, lead to increased TGF-β1secretion in that group.

We found that the preoperative concentration of IL-1α was the lowest among the inflammatory mediators tested. The increased postoperative value of IL-1α was higher and recovered more slowly in the FLEx group than in the SMILE group. IL-1α mRNA and protein are expressed by corneal epithelium [54] but are rarely found in the stroma of normal, unwounded corneas [55]. Once the epithelial barrier is broken, IL-1α production is stimulated. The protein then penetrates the stroma, where it modulates the function of corneal fibroblasts [56]. The release of IL-1α can also trigger the upregulation of various growth factors, including TGF-β1 and TNF-α [57, 58]. The smaller injury to and faster healing of the epithelial surface in SMILE may reduce the expression of IL-1α in tears and accelerate the return to normal level compared to patients who undergo FLEx.

TNF-α and MMP-9, which participate in the mitogen-activated protein kinase (MAPK) signaling pathways in ocular surface epithelial cells, have previously been detected in the corneal epithelium [59] and are upregulated in patients with dry eye [60]. Both proteins are also reported to be involved in corneal wound healing after refractive surgery [61–64]. However, we did not observe any significant difference in TNF-α or MMP-9 levels, either between the two groups or compared to baseline levels. Vesaluoma *et al*. demonstrated a mild increase in TNF-α levels in tear fluid, which was sustained for a short time even after PRK [62]. Mutoh *et al*. stated that fluctuations in MMP-9 activity in the tears were not significantly different from baseline 4 days after PRK. Therefore, we propose that the relatively minor damage to the ocular surface in FLEx and SMILE failed to induce abundant TNF-α and MMP-9 expression. IFN-γ is another vital proinflammatory cytokine secreted by Th1 cells, which causes squamous metaplasia of ocular surface epithelial cells and decreased conjunctival goblet cell density, leading to dry eye [65, 66]. Similarly, no significant change in IFN-γ concentration was detected after either surgery in our study. This may have the same explanation as the lack of changes observed in TNF-α and MMP-9 levels.

In our study, correlation analysis suggested that NGF, TGF-β1, and IL-1α were significantly correlated with OSDI, NI-BUT or FL, which might be associated with damages to the ocular surface resulting from the two different techniques. NGF was found to be correlated with OSDI and NI-BUT in both groups, which was considered as the important inflammatory mediator in FLEx and SMILE surgeries.

The corneal epithelium, stroma and conjunctiva contribute to ocular surface integrity and normal function after refractive surgery. Inflammatory mediators are active and expressed in different parts of the cornea and conjunctiva postoperatively, and some of these enter the tears. In this study, we analyzed the inflammatory markers in collected tear samples, which partially demonstrates the changes in those tissues, but do not include the whole variety of alterations. In addition, we should have observed ocular surface change and inflammatory mediators for a longer follow-up time. Damage to the corneal nerves lasts for at least 6 months after FLEx[20] or SMILE[21, 28, 67] and some inflammatory markers can be detected in tears even at 3 months postoperatively [22]. However, it is not easy to persuade patients to follow up so long and complete all the assessments due to the time consuming examinations and patient logistics. Ideally, the research should have been designed to be double-masked to avoid examiner bias. Unfortunately, it is easy to know what kind of surgery the patients underwent by slit lamp. That is why we couldn't take evaluation in masked manner.

In summary, this prospective study supports our hypothesis that SMILE induces significantly less damage to the ocular surface and causes a more moderate increase in NGF, TGF-β1 and IL-1α than FLEx, which may contribute to the process of ocular surface recovery in early postoperative period. These results show that SMILE is a reliable and minimally invasive refractive surgery for the correction of myopia and astigmatism.

## Supporting Information

S1 TREND Checklist Trend statement checklist. (PDF)

S1 Protocol English protocol of this clinical trial. (PDF)

S2 Protocol Chinese protocol of this clinical trial. (PDF)
