# Supplementary material for: Comparison of Early Changes in Ocular Surface and Inflammatory Mediators between Femtosecond Lenticule Extraction and Small-Incision Lenticule Extraction
Source: PLoS One. 2016 Mar 3;11(3):e0149503. doi: 10.1371/journal.pone.0149503 (PMC4777367; doi:10.1371/journal.pone.0149503)
Supplement: S1 Protocol — (PDF) [file pone.0149503.s002.pdf]

Comparison of Early Changes in Ocular Surface and Inflammatory Mediators between Lenticule Extraction and Small-Incision Lenticule Extraction

Number	2014-005
Sponsor	Xingwu Zhong, MD,PhD (Hainan Eye Hospital, Zhongshan Ophthalmic Center, Sun Yat-sen University)
Funding Oranization	National Natural Science Foundation of China (81371046)<br>Science and Technology Planning Project of Guangdong Province （2013B090200057）
Principal Investigator	Xingwu Zhong MD,PhD<br>Telephone: +86(757)68628481<br>Fax: +86(757)68628587<br>E-mail: **zhongxwu@mail.sysu.edu.cn**
Primary Working Investigators	Xingwu Zhong, MD, PhD<br>Chi Zhang, MD

Approval:

______________________________ ________________

PI or Sponsor Signature(Name and Title) *Date*

PROTOCOL SYNOPSIS

**TITLE**	Comparison of Early Changes in Ocular Surface and Inflammatory Mediators between Lenticule Extraction and Small-Incision Lenticule Extraction
**SPONSOR**	Xingwu Zhong, MD,PhD
**FUNDING ORGANIZATIONS**	National Natural Science Foundation of China (81371046)<br>Science and Technology Planning Project of Guangdong Province （2013B090200057）
**RATIONALE**	This first all-in-one FS-laser system was designed to perform the refractive lenticule extraction (ReLEx) precedures, femtosecond lenticule extraction (FLEx) and small-incision lenticule extraction (SMILE).In FLEx, a corneal flap is created by the FS laser (similar to LASIK) and lifted, allowing lenticule removal[1-3]. For SMILE, a truly flapless procedure, only a small—2-4mm—incision is made, through which the lenticule is removed. Multiple etiologies contribute to this ocular surface disruption, including the flap creation and stromal ablation involved in previous refractive surgery techniques, such as: disable tear film, damage of the corneal nerve and postoperative inflammatory mediator.For both FLEx and SMILE, stromal ablation has been replaced by refractive lenticule removal. In terms of corneal flap formation, FLEx still requires an epithelial-stromal flap, while SMILE employs only a small incision to extract the lenticule. Hence, we hypothesize that SMILE will have less effect on patients' ocular surface markers and inflammatory mediators, compared to FLEx.To test our hypothesis, we conducted a prospective clinical study in patients who underwent FLEx or SMILE. Ocular surface parameters and inflammatory mediators were assessed and compared between the different types of surgery.
**STUDY DESIGN**	This is a prospective, non-randomly study
**PRIMARY OBJECTIVE**	Determine the changes of central corneal sensitivity, Schirmer I test (SIT), noninvasive tear breakup time (NI-TBUT), tear meniscus height, corneal fluorescein (FL) staining, ocular surface disease index (OSDI) and tear concentrations of interleukin-1α (IL-1α), tumor necrosis factor-α (TNF-α), nerve growth factor (NGF), interferon-γ (IFN-γ), transforming growth factor-β1 (TGF-β1) and matrix metalloproteinase-9 (MMP-9) of the two surgeries preoperatively and postoperatively.
**SECONDARY OBJECTIVES**	Determine the correlation between inflammatory mediators and ocular surface changes.
**NUMBER OF SUBJECTS**	41 patients in total

**INCLUSION CRITERIA**	1. Minimum age of 18 years(range from 18 year to 25 years).<br>2. Corneal thickness more than 500 μm and calculated residual stromal bed after treatment greater than 300 μm.<br>3. Corneal thickness more than 500 μm and calculated residual stromal bed after treatment greater than 300 μm.<br>4. Preoperative cylindrical equivalent refraction between -0.25 D and -1.50 D.<br>5. Preoperative corneal curvature from 41.0 D to 46.0 D with a regular topographic pattern.<br>6. monocular best corrected visual acuity of 20/20 or better and stable refractive error (less than 0.5 D change) for 24 months before surgery.
**EXCLUSION CRITERIA**	1. Systemic disease that contraindicated the surgery (such as diabetes, glaucoma and systemic collagen vascular disease).<br>2. Corneal abnormality or disease.<br>3. A history of tear supplement usage.<br>4. Contact lens wear during the past year.
**GROUPS**	Group 1: patients underwent FLEx surgery.<br>Group 2: patients underwent SMILE surgery.
**DURATION OF SUBJECT PARTICIPATION AND DURATION OF STUDY**	Subjects will be on study for up to 2 months<br>Screening: 2-4weeks<br>Treatment: 1day<br>Follow-up: 1day, 1week and1month (1 month in total)<br>The total duration of the study is expected to be 9 months.
**STATISTICS PLAN**	Data were analyzed using SPSS 19.0 software (SPSS, Chicago, IL, USA). Paired samples t-test (normally distributed data) or Wilcoxon test (non-normally distributed data) was used to determine significant differences between pre- and post-operative measurements in each group. Comparisons between the two groups were performed by Independent samples t-test (normally distributed data) or Mann-Whitney U test (non-normally distributed data). Pearson or Spearman rank correlation was used to analyzed for the correlations between tear inflammatory mediators and ocular surface parameters. *P* value of less than 0.05 was considered statistically significant. Data are presented as mean ± SD.

1 BACKGROUND

The use of femtosecond (FS) laser has become one of the most significant technological advancements in refractive surgery. A breakthrough FS laser-assisted myopic and myopic astigmatic correction procedure can now be performed using a prototype of the VisuMax femtosecond system. This first all-in-one FS-laser system was designed to perform the refractive lenticule extraction (ReLEx) precedures, femtosecond lenticule extraction (FLEx) and small-incision lenticule extraction (SMILE). In FLEx, a corneal flap is created by the FS laser (similar to LASIK) and lifted, allowing lenticule removal. For SMILE, a truly flapless procedure, only a small—2-4mm—incision is made, through which the lenticule is removed.

Ocular surface disruption during corneal refractive surgery is commonly considered to be closely related to the development of dry eye. Multiple etiologies contribute to this ocular surface disruption, including the flap creation and stromal ablation involved in previous refractive surgery techniques. Corneal nerve damage has been considered the main cause of dry eye, due to disrupted afferent sensory nerves, reduced blink reflex, and increased tear evaporation leading to tear film instability. In addition, postoperative inflammatory mediator fluctuations are also a key factor related to ocular surface damage. Extensive research has described the effects of cytokines, chemokines and growth factors in modulating corneal wound healing, cell migration, and apoptosis on the ocular surface after refractive surgery.

2 STUDY RATIONALE

This first all-in-one FS-laser system was designed to perform the refractive lenticule extraction (ReLEx) precedures, femtosecond lenticule extraction (FLEx) and small-incision lenticule extraction (SMILE).In FLEx, a corneal flap is created by the FS laser (similar to LASIK) and lifted, allowing lenticule removal[1-3]. For SMILE, a truly flapless procedure, only a small—2-4mm—incision is made, through which the lenticule is removed. Multiple etiologies contribute to this ocular surface disruption, including the flap creation and stromal ablation involved in previous refractive surgery techniques, such as: disable tear film, damage of the corneal nerve and postoperative inflammatory mediator.For both FLEx and SMILE, stromal ablation has been replaced by refractive lenticule removal. In terms of corneal flap formation, FLEx still requires an epithelial-stromal flap, while SMILE employs only a small incision to extract the lenticule. Hence, we hypothesize that SMILE will have less effect on patients' ocular surface markers and inflammatory mediators, compared to FLEx.To test our hypothesis, we conducted a prospective clinical study in patients who underwent FLEx or SMILE. Ocular surface parameters and inflammatory mediators were assessed and compared between the different types of surgery.

2.1 Risk/Benefit Assessment

There are no adverse events reported in the literature do date. The risks associated with the research is indicated as following:

2.1.1 Risk of dry eye

The patients may experience dry eye after the refractive surgery. We will exclude the patients who already have dry eye in order to avoid more potential sever dry eye syndrome postoperatively.

2.1.2 Risk of overcorrections or undercorrections

We will make a appropriate surgery strategy according to patients' refractive status, best corrected visual acuity and demands.

2.1.3 Risk of vision returning

The risk of vision returning for patients with high myopia may be higher than patients with medium and low myopia after surgery

3 STUDY OBJECTIVES

3.1 Primary Objective

3.1.1.Determine the changes of ocular surface including central corneal sensitivity, Schirmer I test (SIT), noninvasive tear breakup time (NI-TBUT), tear meniscus height, corneal fluorescein (FL) staining, ocular surface disease index (OSDI) of the two surgeries preoperatively and postoperatively

3.1.2.Evaluation the changes of tear concentrations of interleukin-1α (IL-1α), tumor necrosis factor-α (TNF-α), nerve growth factor (NGF), interferon-γ (IFN-γ), transforming growth factor-β1 (TGF-β1) and matrix metalloproteinase-9 (MMP-9) of the two surgeries preoperatively and postoperatively.

3.2 Secondary Objective

Determine the correlation between inflammatory mediators and ocular surface changes.

4 STUDY DESIGN

This is a single center, prospective and non-randomly study. 41 patients are planned to be enrolled. They are divided into two groups. One group will undergo the FLEx surgery, the other group will undergo the SMILE surgery. The ocular surface parameters and concentration of tear inflammatory mediators will be assessed at baseline, 1 day,1 week and 1 month postoperatively. Subjects who meet all inclusion criteria and none of the exclusion criteria will be entered into the study.

1. Minimum age of 18 years(range from 18 year to 25 years).

2. Corneal thickness more than 500 µm and calculated residual stromal bed after treatment greater than 300 µm.

3. Corneal thickness more than 500 µm and calculated residual stromal bed after treatment greater than 300 µm.

4. Preoperative cylindrical equivalent refraction between -0.25 D and -1.50 D.

5. Preoperative corneal curvature from 41.0 D to 46.0 D with a regular topographic pattern.

5 SUBJECT SELECTION

5.1 Study Population

Subjects with a diagnosis of myopia and/or astigmatism who meet the inclusion and exclusion criteria will be qualified to participate in this study.

5.2 Inclusion Criteria

1. Minimum age of 18 years(range from 18 year to 25 years).

2. Corneal thickness more than 500 µm and calculated residual stromal bed after treatment greater than 300 µm.

3. Corneal thickness more than 500 µm and calculated residual stromal bed after treatment greater than 300 µm.

4. Preoperative cylindrical equivalent refraction between -0.25 D and -1.50 D.

5. Preoperative corneal curvature from 41.0 D to 46.0 D with a regular topographic pattern.

5.3 Exclusion Criteria

1. Systemic disease that contraindicated the surgery (such as diabetes, glaucoma and systemic collagen vascular disease).

2. Corneal abnormality or disease.

3. A history of tear supplement usage.

4. A history of contact lens wear during the past year.

6 CONCURRENT MEDICATIONS

Standard postoperative treatment consisted of 0.3% tobramycin/dexamethasone (TobraDex, Alcon) eyedrops, 0.5% levofloxacin (Cravit, Santen) eyedrops and sodium hyaluronate (HYCOSAN,URSAPHARM Arzneimittel GmbH) four times a day for one week. Tobradex and Cravit were suspended after one week, but artificial tear eyedrops were applied as required until one month.

7 STUDY PROCEDURES AND GUIDELINES

All patients provided written consent to participate in the study and finished all postoperative follow-up visits. All surgeries were performed by the same experienced surgeon (Xingwu Zhong) following standard procedures under topical anesthesia. One eye of each subject was selected randomly for statistical analysis.

7.1 Surgery Procedure

7.1.1 FLEx

The VisuMax femtosecond laser system (Carl Zeiss Meditec AG, Jena, Germany) with a 500 kHz repetition rate will be used to perform FLEx surgery. Four femtosecond incisions will be created in succession: the posterior surface of the refractive lenticule (spiral in), the lenticule border, the anterior surface of the refractive lenticule (spiral out), and the corneal flap in the superior region. After the suction is released, the flap is opened using a thin, blunt spatula and the free refractive lenticule is subsequently grasped with a forceps and extracted, after which the flap is repositioned carefully.

The planned flap thickness with superior hinge and 50 degrees in cordal length is 120 μm. The flap diameter is 7.5 μm and the lenticule diameter 6.5 mm. The optical zone size is 6.5 μm. The spot spacing and tracking spacing is 4.5 μm for the lenticule and 2.0 μm for the lenticule side cut. The energy of the femtosecond laser is 140nJ.

7.1.2 SMILE

ReLEx SMILE surgery will be also performed using the Visu Max femtosecond laser system with a 500 kHz repetition rate. The only difference from the ReLEx FLEx procedure is to make a small incision in the last step, instead of creating a corneal flap. Once the four incisions is created, suction is released automatically and the anterior and posterior refractive surfaces are separated by a thin, blunt spatula. The refractive lenticule is extracted through the incision using forceps.

The optical zone size was 6.5 mm. The anterior lenticule surface was 120 μm deep. The small incision was located in the 120º position, with 50 μm cordal length (the side-cut incision with a circumferential length of 4.0–5.0 mm and angle of 90 º). The spot spacing and tracking spacing is 4.5 mm for the lenticule, 2.0 mm for the lenticule side cut, 3.0 mm for the small incision and 2.0 mm for the small incision side cut. The energy of the femtosecond laser is also 140nJ.

7.2 Treatment after Surgery

Standard postoperative treatment consisted of 0.3% tobramycin/dexamethasone (TobraDex, Alcon) eyedrops, 0.5% levofloxacin (Cravit, Santen) eyedrops and sodium hyaluronate (HYCOSAN,URSAPHARM Arzneimittel GmbH) four times a day for one week. Tobradex and Cravit were suspended after one week, but artificial tear eyedrops were applied as required until one month.

7.3 Clinical Assessments

7.3.1 Corneal Central Sensitivity (CCS)

Corneal central sensitivity is measured with a Cochet-Bonnet esthesiometer (Luneau, Paris, France). This instrument consists of a nylon monofilament that is 60 mm in length and with diameter of 0.12 mm. The instrument is advanced perpendicular to the central surface of the cornea until contact between the instrument and the cornea is made. If the patient feels the filament, the response is defined positive. corneal central sensitivity is tested three times with each filament length, and the length of the filament is sequentially reduced from 60 mm in 5-mm steps. At least two positive responses among three attempts are considered a positive result at each filament length. The longest filament length that resulted in a positive result is considered the corneal threshold. This evaluation will just be performed at baseline ,1 week and 1 month after surgery in order to avoid the potential for corneal damage at 1 day postoperatively.

7.3.2 Schirmer I Test (SIT)

The Schirmer test without anesthesia (S1T) for tear secretion function will be performed by inserting a 30-mm Schirmer tear test strip (Jingming, Tianjing, China) into the inferior fornix at the junction of the middle and lateral thirds of the lower eyelid margin. Schirmer test strips will remain in place for 5 minutes with the eyes closed. The extent of wetting will be subsequently measured according to the scale provided by the manufacturer. Potential scores ranged from 0 to 30 mm, with lower scores indicating greater tear production abnormalities. This evaluation will be performed at baseline, 1day, 1 week and 1 month after surgery.

7.3.3 Corneal Fluorescein Staining (FL)

Upper, lower, nasal, temporal and central quadrants were used. Superficial punctate keratopathy (SPK) in the cornea was scored from 0 to 3 in each quadrant: 0, no staining in the cornea; 1, ＜5 punctuate stains; 2, ＞5 punctuate stains but ＜10; and 3, ＞10 or filamentous staining detected. The total number was obtained by adding the scores of the four quadrants for each eye (0–15). This evaluation will just be performed at baseline ,1 week and 1 month after surgery in order to avoid the potential for corneal damage at 1 day postoperatively.

7.3.4 Noninvasive Tear Breakup Time (NI-BUT)

Four IR diodes were set on the Keratograph 5 (Oculus, Wetzlar, Germany) and arranged in two pairs, located one above the other. The red ring illumination used for corneal topography was deactivated; this ensured a dark background for the examination. An illuminated ring pattern was then projected onto the cornea. At the start of the recording, the subject will be asked to blink his or her eyes three times and then to keep them open as long as possible. Irregularities in the reflected image will be observed closely. Special attention will be given to distortions and gaps in individual rings and the time such deviations from the original ring pattern took to occur. The examination will be recorded on video. This evaluation will be performed at baseline, 1day, 1 week and 1 month after surgery.

7.3.5 Tear Meniscus Height (TMH)

The subject will be asked to blink his or her eyes once ,then the image of tear meniscus height will be captured by a Keratograph 5 (Oculus, Wetzlar, Germany).After that tear meniscus height will be measured at the centre of the lower lid margin. This performance will be taken three times and the average value will be recorded. This evaluation will be performed at baseline, 1day, 1 week and 1 month after surgery.

7.3.6 Ocular Surface Disease Index (OSDI)

The OSDI questionnaire is used to quantify the dry eye symptoms. Subjects will be asked questions regarding the dry eyesymptoms that they had experienced; the OSDI questions is drawn from 3 different subscales: ocular symptoms, vision-related functions, and environmental triggers. Each answer is scored on a 4-point scale from zero (indicating no problems) to four (indicating a significant problem). Responses to all of the questions are combined to generate a composite OSDI score that ranges from 0 to 100, with higher OSDI scores indicating more severe symptoms. Symptoms of dry eye, such as dryness,

burning, foreign body sensation, stabbing pain, photophobia, and visual fluctuations, are also noted. This evaluation will be performed at baseline, 1day, 1 week and 1 month after surgery.

7.4 Clinical Laboratory Measurements

7.4.1 Tear Collection

A nonstimulated tear sample was collected by using disposable 5-mL microcapillaries (Microcaps 5 mL; Drummond Scientific, Broomall, PA). Tear collection was performed from the inferior marginal region without irritation of the cornea, conjunctiva or lid margin. A 20μl sample was obtained, transferred to a 0.5 ml microtube, and stored at -80 °C until processing.

7.4.2 Inflammatory Mediators Assay

Interleukin-1α (IL-1α), tumor necrosis factor-a (TNF-α), nerve growth factor (NGF), interferon-γ (IFN-γ), transforming growth factor-β1 (TGF-β1) and matrix metalloproteinase-9 (MMP-9) in collected tears were measured by a Quantibody Human Inflammation Array I kit (RayBiotech, Inc. Norcross, GA) according to the manufacturer's instructions. In brief, antibodies against the inflammatory mediators were spotted onto the cytokine array. After incubation with tear samples for 2 hours, biotin-conjugated secondary antibodies were added for 1 hour. Then the Cy3 dye-conjugated streptavidin was added for another 1 hour. The signals were captured by GenePix 4000B (Bio-Rad Laboratories, Hercules, CA) and analyzed by Quantibody® Q-Analyzer software (RayBiotech, Inc. Norcross, GA).The concentration was quantified according to the standard curves generated from standards provided by the manufacturer.

8. ADVERSE EXPERIENCE REPORTING AND DOCUMENTATION

Dr. Xingwu Zhong should be contacted directly at this number to report medical concerns or questions regarding safety.
Phone: +86(757)68628481

9. DISCONTINUATION AND REPLACEMENT OF SUBJECTS

9.1 Withdrawal of Subjects from the Study may be withdrawn from the study at any time if the subject, the investigator, or the Sponsor feels that it is not in the subject's best interest to continue. All subjects are free to withdraw from participation at any time, for any reason, specified or unspecified, and without prejudice. Reasonable attempts will be made by the investigator to provide a reason for subject withdrawals. The reason for the subject's withdrawal from the study will be specified in the subject's source documents.

9.2 Replacement of Subjects who withdraw from the study will be replaced.

10. PROTOCOL VIOLATIONS

A protocol violation occurs when the subject fails to adhere to significant protocol requirements affecting the inclusion, exclusion, subject safety and primary endpoint criteria. Protocol violations for this study include, but are not limited to, the following:
1.Failure to meet inclusion/exclusion criteria
2.Use of a prohibited concomitant medication

11. ADMINISTRATIVE, ETHICAL, REGULATORY CONSIDERATIONS

The study will be conducted according to the Declaration of Helsinki. To maintain confidentiality, all laboratory specimens, evaluation forms, reports and other records will be identified by a coded number and initials only. All study records will be kept in a locked file cabinet and code sheets linking a patient's name to a patient identification number will be stored separately. Clinical information will not be released without written permission of the subject. The Investigator must also comply with all applicable privacy regulations .

14.1 The Ethics Committees

The protocol and consent form will be reviewed and approved by the IEC of each participating center prior to study initiation. Serious adverse experiences regardless of causality will be reported to the IEC in accordance with the standard operating procedures and policies of the IEC, and the Investigator will keep the IEC informed as to the progress of the study. The Investigator will obtain assurance of IEC compliance with regulations. Protocol and/or informed consent modifications or changes may not be initiated without prior written IEC approval except when necessary to eliminate immediate hazards to the patients or when the change(s) involves only logistical or administrative aspects of the study. Such modifications will be submitted to the IEC and written verification that the modification was submitted and subsequently approved should be obtained. The IEC must be informed of revisions to other documents originally submitted for review; serious and/or unexpected adverse experiences occurring during the study in accordance with the standard operating procedures and policies of the IEC; new information that may affect adversely the safety of the patients of the conduct of the study; an annual update and/or request for re-approval; and when the study has been completed.

14.2 Protocol Amendments

Protocol amendments cannot be implemented without prior written IEC approval except as necessary to eliminate immediate safety hazards to patients.

14.3 Informed Consent Form

Informed consent will be obtained in accordance with the Declaration of Helsinki and local regulations. The consent form generated by the Investigator must be acceptable to the Sponsor and be approved by the IEC. The Investigator will send an IEC-approved copy of the Informed Consent Form to the Sponsor (or designee) for the study file. A properly executed, written, informed consent will be obtained from each subject prior to entering the subject into the trial. Information should be given in both oral and written form and subjects must be given ample opportunity to inquire about details of the study.
