# Supplementary material for: Comparison of Early Changes in Ocular Surface and Inflammatory Mediators between Femtosecond Lenticule Extraction and Small-Incision Lenticule Extraction
Source: PLoS One. 2016 Mar 3;11(3):e0149503. doi: 10.1371/journal.pone.0149503 (PMC4777367; doi:10.1371/journal.pone.0149503)
Supplement: S2 Protocol — (PDF) [file pone.0149503.s003.pdf]

# 比较 FLEx 和 SMILE 术后早期眼表改变及炎症介质变化

| | |
|---|---|
| 项目编号 | 2014-005 |
| 赞助人 | 钟兴武， MD, PhD （中山大学中山眼科中心海南省眼科医院） |
| 资金来源 | 中国国家自然科学基金项目(81371046)<br>广东省科技计划项目（2013B090200057） |
| 项目负责人 | 钟兴武， MD, PhD<br>电话：+86(757)68628481<br>传真: +86(757)68628587<br>E-mail：**zhongxwu@mail.sysu.edu.cn** |
| 主要研究人员 | 钟兴武，MD, PhD<br>张弛，MD |

**批准:**

_________________________________ ________________

*项目负责人或者资助人签名* *日期*

# 研究方案大纲

| | |
|---|---|
| **题目** | 比较 FLEx 和 SMILE 术后的早期眼表改变及炎症介质变化 |
| **资助人** | 钟兴武，MD, PhD<br>中山大学中山眼科中心海南省眼科医院 |
| **资金来源** | 中国国家自然科学基金项目(81371046)<br>广东省科技计划项目（2013B090200057） |
| **研究原理** | 飞秒基质透镜切除术（FLEx）和小切口角膜微透镜取出术（SMILE）都是第一次采用一体化飞秒激光系统来执行屈光微透镜取出的程序。FLEx 是应用飞秒激光制作角膜瓣后（类似于 LASIK），取出透镜式片状角膜组织。而 SMILE 是真正做到无角膜瓣切削过程，只需 2-4mm 的小切口，使其切削的透镜从小切口取出。多种病因常导致眼表功能破坏，包括屈光手术中所涉及的角膜瓣制作和基质消融技术，如：泪膜功能破坏，角膜感觉神经损伤和术后炎症反应。FLEx 和 SMILE 中都采用屈光透镜取出术来代替以前的角膜基质消融术。FLEx 仍需要制作角膜上皮基质瓣，而 SMILE 仅需一个小切口取出切削透镜。因此，我们假设相较于 FLEx，SMILE 能减少病人的眼表功能异常的症状和炎症反应。我们在接受了 FLEx 或 SMILE 手术的病人中进行了前瞻性临床研究来证实我们的设想。通过术后的眼表参数的测量和炎症介质反应程度来评估比较两组病人的情况。 |
| **研究设计** | 前瞻性、非随机性研究 |
| **主要研究目标** | 对于两组病人的术前及术后测定中央角膜的敏感性变化，基础泪液分泌实验 I（Schirmer I test，SIT），无创新泪膜破裂时间（noninvasive tear breakup time ，NI-TBUT），泪液凹面高度，角膜荧光素染色，眼表疾病指数（ocular surface disease index，OSDI）和泪液中相关细胞因子浓度的测定，包括细胞白介素- 1α（interleukin-1α ，IL-1α），肿瘤坏死因子-α（tumor necrosis factor-α，TNF-α），神经生长因子（nerve growth factor ，NGF），干扰素-γ（interferon-γ ，IFN-γ），转化生长因子-β1（transforming growth factor-β1，TGF-β1）和基质金属蛋白酶-9（matrix metalloproteinase-9 ，MMP-9）。 |
| **次要研究目标** | 研究炎性介质和眼表变化之间的相关性。 |
| **研究对象** | 共 41 个病人 |

| | |
|---|---|
| **纳入标准** | 1.年满 18 岁（18 岁-25 岁）；<br>2.角膜厚度大于 500μm，并且计算剩余角膜基质床厚度大于 300μm；<br>3.术前散光屈光介于-0.25 D 至-1.50 D 之间；<br>4.术前角膜地形图规则，角膜曲率介于 41.0 D 至 46.0 D 之间；<br>5.最佳矫正视力为 20/20 或者更好，至少术前 24 个月至今具有稳定的屈光不正（屈光度数变化小于 0.5D）。 |
| **排除标准** | 1.具有手术禁忌的全身系统性疾病（如糖尿病，青光眼和系统性胶原血管病变等）；<br>2.角膜异常或角膜病变；<br>3.具人工泪液使用史；<br>4.过去 1 年有隐形眼镜佩戴史。 |
| **分组** | 1 组：FLEx 的手术病人<br>2 组：SMILE 的手术病人 |
| **观察时间** | 研究持续观察 2 个月<br>筛选时间：2-4 周<br>治疗时间：1 天<br>随访时间：第 1 天，第 1 周，第 1 个月（总共 1 月）<br>研究时间预计为 9 个月。 |
| **统计方法** | 数据分析采用 SPSS 19.0 软件（SPSS, Chicago, IL, USA）。用配对 T 检验或者 Wilcoxon 检验检测两组病人术前术后的组内差异性，单样本 T 检验或 Mann-Whitney U 检验检测两组病人的组间差异性。 Pearson 检验或 Spearman 等级相关检验检测泪液炎症介质及眼表相关参数的相关性。$P<0.05$ 具有统计学意义。数据均采用 $\bar{x}\pm SD$ 表示。 |

## 1 研究背景

使用飞秒激光的屈光手术已成为目前最先进的技术。而具有突破性进展的是以德国蔡司全飞秒激光（VisuMax）为原型的操作系统，使用飞秒激光矫正近视和近视性散光。此操作系统是第一个一体化的飞秒激光系统，用于屈光微透镜取出术（ReLEx）、飞秒基质透镜切除术（FLEx）和小切口角膜微透镜取出术（SMILE）。FLEx 需要用飞秒激光制作角膜瓣后（类似于 LASIK），取出透镜式片状角膜组织。而 SMILE 是真正做到无角膜瓣切削过程，只需 2-4mm 的小切口，使其切削的透镜从小切口取出。

通常认为在屈光手术时发生的眼表功能破坏与干眼的发生与密切联系。有多种原因导致眼表功能紊乱，包括屈光手术中角膜瓣的制作和基质消融技术。角膜神经损伤被认为是干眼的主要原因，由于中断了感觉传入神经，瞬目反射减弱，增加了泪液蒸发，最后导致泪膜功能不稳定。此外，术后炎性介质反应也是导致眼表功能破坏的重要因素。已有大量的研究表明许多细胞因子也对眼屈光手术后的眼表功能存在影响，如趋化因子和生长因子可调节角膜伤口愈合、细胞迁移及细胞凋亡等。

**2 研究原理**

操作系统是第一个一体化的飞秒激光系统，用于屈光微透镜取出术（ReLEx）、飞秒基质透镜切除术（FLEx）和小切口角膜微透镜取出术（SMILE）。FLEx 需要用飞秒激光制作角膜瓣后（类似于 LASIK），取出透镜式片状角膜组织。而 SMILE 是真正做到无角膜瓣切削过程，只需 2-4mm 的小切口，使其切削的透镜从小切口取出。多种病因常导致眼表功能破坏，包括屈光手术中所涉及的角膜皮瓣制作和基质消融技术，例如：泪膜功能破坏，角膜感觉神经损伤和术后炎症反应。FLEx 和 SMILE 中都采用屈光透镜取出术来代替以前的角膜基质消融术。FLEx 仍需要制作角膜上皮基质瓣，而 SMILE 仅需一个小切口取出切削透镜。因此，我们假设相较于 FLEx，SMILE 能减少病人的眼表功能异常的症状和炎症反应。我们在接受了 FLEx 或 SMILE 手术的病人中进行了前瞻性临床研究来证实我们的设想。通过术后的眼表参数和炎症介质反应程度来评估比较两组病人的情况。

**2.1 有利及不利因素**

到目前为止没有任何关于此飞秒技术的不良报道。与研究相关的风险因素如下：

**2.1.1 干眼的发生**

屈光手术后的病人可能会发生干眼。我们研究前会排除已有干眼症的病人，以此避免术后发生更加严重的干眼综合征。

**2.1.2 屈光度数过矫正或矫正不足**

研究时会根据病人的屈光情况，最佳视敏度及具体日常要求来制定合适的手术方案。

#### 2.1.3 视力回退的风险

高度近视的病人相较于中低度近视的病人出现视力回退的风险更高。

## 3 研究目标

### 3.1 主要目标

3.1.1 对于两组病人的术前及术后测定中央角膜的敏感性变化，基础泪液分泌实验 I（Schirmer I test，SIT），无创新泪膜破裂时间（noninvasive tear breakup time ，NI-TBUT），泪液凹面高度，角膜荧光素染色，眼表疾病指数（ocular surface disease index，OSDI）。

3.1.2 评估两组病人术前、术后泪液中相关细胞因子浓度的测定，包括细胞白介素- 1α（interleukin-1α ，IL-1α），肿瘤坏死因子-α（tumor necrosis factor-α，TNF-α），神经生长因子（nerve growth factor ，NGF），干扰素-γ（interferon-γ ，IFN-γ），转化生长因子-β1（transforming growth factor-β1，TGF-β1）和基质金属蛋白酶-9（matrix metalloproteinase-9 ，MMP-9）。

### 3.2 次要目标

研究炎性介质和眼表变化之间的相关性。

## 4 研究设计

研究采用单样本前瞻性非随机性试验。41 名病人为计划安排。他们被分为 2 组。1 组接受 FLEx 手术，另 1 组接受 SMILE 手术。手术前及术后第 1 天、第 1 周、第 1 月的眼表参数及炎症介质浓度均在同一基线、标准测定。研究对象均符合所有纳入标准，无排除标准中情况。

**纳入标准：**

1.年满 18 岁（18 岁-25 岁）；

2.角膜厚度大于 500μm，并且计算剩余角膜基质床厚度大于 300μm；

3.术前散光屈光介于-0.25 D 至-1.50 D 之间；

4.术前角膜地形图规则，角膜曲率介于 41.0 D 至 46.0 D 之间；

5.最佳矫正视力为20/20或者更好，至少术前24个月至今具有稳定的屈光不正（屈光度数变化小于 0.5D）。

**排除标准：**

1.手术禁忌的全身系统性疾病（如糖尿病，青光眼和系统性胶原血管病变等）；

2.角膜异常或角膜病变；

3.具人工泪液使用史；

4.过去 1 年有隐形眼镜佩戴史。

## 5 研究对象选取

### 5.1 研究人群

研究对象均符合所有纳入标准，无排除标准中情况。

### 5.2 纳入标准：

1.年满 18 岁（18 岁-25 岁）；

2.角膜厚度大于 500μm，并且计算剩余角膜基质床厚度大于 300μm；

3.术前散光屈光介于-0.25 D 至-1.50 D 之间；

4.术前角膜地形图规则，角膜曲率介于 41.0 D 至 46.0 D 之间；

5.最佳矫正视力为20/20或者更好，至少术前24个月至今具有稳定的屈光不正（屈光度数变化小于 0.5D）。

### 5.3 排除标准：

1.手术禁忌的全身系统性疾病（如糖尿病，青光眼和系统性胶原血管病变等）；

2.角膜异常或角膜病变；

3.具人工泪液使用史；

4.过去 1 年有隐形眼镜佩戴史。

## 6 术后药物治疗

术后病人均用 0.3%妥布霉素地塞米松滴眼液（TobraDex, Alcon）、0.5%左眼氟沙星眼液（Cravit, Santen）和玻璃酸钠眼液（HYCOSAN, URSAPHARM

Arzneimittel GmbH）治疗，使用频率均为 1 天 4 次（qid），1 周后停用妥布霉素地塞米松滴眼液和左眼氟沙星眼液，人工泪液持续用 1 个月。

## 7 研究程序和指导

所有的病人均签署同意此项研究的协议书，也按时完成了术后的随访。研究中所有的手术都是由具备经验的外科医生（钟兴武教授）在局部麻醉下按照标准手术方法实施。每个病人随机选取一个眼作统计分析。

### 7.1 手术方法

#### 7.1.1 飞秒基质透镜切除术（FLEx）

FLEx 手术采用德国蔡司全飞秒系统（Carl Zeiss Meditec AG, Jena, Germany）进行扫描切割，频率为 500 kHz。4 个飞秒切口按照以下顺序：基质内透镜后表面（内螺旋），微透镜侧切边界，微透镜的前表面（外螺旋），最优部位的角膜瓣。释放压力吸入，使用薄、钝性的显微刮刀掀开角膜瓣，用显微镊取出屈光微透镜，最后小心使角膜瓣复位。

设计的角膜瓣厚度具有完好的转轴和中轴长度为 120μm ，弧度为 50°。角膜瓣半径为 7.5μm，微透镜直径为 6.5μm，光学区半径为 6.5μm。微透镜的点间距和线间距为 4.5μm，微透镜侧切为 2.0μm。飞秒激光能力为 140nJ。

#### 7.1.2 小切口角膜微透镜取出术（SMILE）

SMILE 手术也是采用德国蔡司全飞秒系统（Carl Zeiss Meditec AG, Jena, Germany）进行扫描切割，频率为 500 kHz。与 FLEx 不同的是手术最后一步，用小切口代替制作角膜瓣。完成前 4 步切削后，释放压力吸入，使用薄、钝性的刮刀分离前后切削面，用显微镊取出屈光微透镜。

光学半径为 6.5 mm，前微透镜表面深达 120 μm。小切口位于 120 º位置，弧度 50 º（切口长度为 4-5 mm，90 º角）。微透镜的点间距和线间距为 4.5μm，微透镜侧切为 2.0μm。小切口为 3mm，小切口侧边为 2mm，飞秒激光能力为 140nJ。

### 7.2 术后治疗

术后病人均用 0.3%妥布霉素地塞米松滴眼液（TobraDex, Alcon）、0.5%左眼氟沙星眼液（Cravit, Santen）和玻璃酸钠眼液（HYCOSAN, URSAPHARM Arzneimittel GmbH）治疗，使用频率均为 1 天 4 次（qid），1 周后停用妥布霉素地塞米松滴眼液和左眼氟沙星眼液，人工泪液持续用 1 个月。

## 7.3 临床评估

### 7.3.1 中央角膜敏感度（Corneal Central Sensitivity，CCS）

用 Cochet-Bonnet esthesiometer (Luneau, Paris, France)测量中央角膜敏感度。该仪器包括的尼龙丝长度为 60㎜，直径 0. 12㎜。这个仪器垂直于角膜表面测量，可以接触到中央角膜表面。如果病人感觉到尼龙丝，则判断为阳性反应。用不用长度的尼龙丝测量，每次重复 3 次。尼龙丝长度从 60㎜ 以 5㎜ 往下递减。至少两次出现阳性反应才判断为阳性反应。最长的尼龙丝测量阳性结果为角膜阈值。测量结果采用统一标准，在 1 周、1 月测量，为避免术后角膜损伤，术后 1 天不测量。

### 7.3.2 泪液分泌试验（Schirmer I Test，SIT）

泪液分泌实验在无麻醉下进行。用 30mm 泪液测试条（Jingming，Tianjin, China）放入下睑穹窿内侧 1/3。放入测试条后，闭眼 5min。湿润程度根据制造商提供的说明进行测量。计量范围为 0-30mm，程度越低说明泪液分泌越有可能异常。测量结果采用统一标准，在术后 1 天、1 周、1 月进行。

### 7.3.3 角膜荧光染色（Corneal Fluorescein Staining ，FL）

能观察到角膜上、下、鼻、颞侧的染色。点状角膜病变（Superficial punctate keratopathy ，SPK）在每个象限的分为 0-3 分：0 分为无染色；1 分为小于 5 个染色点；2 分为大于 5 个小于 10 个染色点；3 分为大于 10 个染色或出现丝状染色。四个象限的得分相加为每眼的总分。测量结果采用统一标准，在 1 周、1 月测量，为避免术后角膜损伤，术后 1 天不测量。

### 7.3.4 无创性测量泪膜破裂时间（Noninvasive Tear Breakup Time，NI-BUT）

测量时的暗室环境由四个红外二极管成双设置于 Keratograph 5 (Oculus, Wetzlar, Germany)上激发形成。选择环状光线投射在角膜上。开始测量前，受试者先眨眼 3 次，再保持开眼。密切观察光线出现缺损、变形的时间，并录像记录。测量结果采用统一标准，在 1 周、1 月测量，为避免术后角膜损伤，术后 1 天不测量。

#### 7.3.5 泪河高度测量（Tear Meniscus Height ， TMH）

研究对象在测试前眨眼数次，泪河高度图像由 Keratograph 5(Oculus, Wetzlar, Germany)拍照。高度从泪河中心的最低点边缘测量。测量重复 3 次取平均值。测量结果采用统一标准，在术后 1 天、1 周、1 月进行。

#### 7.3.6 眼表疾病指数（Ocular Surface Disease Index ， OSDI）

OSDI 问卷是用来量化干眼症状的。问卷根据干眼症状提问，病人是否经历过干眼的症状等。OSDI 问卷包含 3 部分：眼部症状、相关的视力问题、环境因素。每个问题都有评分，0-4 分不等，0 分为无症状，4 分为有明显症状。回答所有问题会产生 0-100 分不等，分数越高说明干眼症状更明显。干眼的症状如眼干、灼热感、异物感、眼痛、畏光和视力波动等。测量结果采用统一标准，在术后 1 天、1 周、1 月进行。

### 7.4 临床检验科检测

#### 7.4.1 泪液收集

使用一次性的 5ml 微细管（Microcaps 5 mL; Drummond Scientific, Broomall, PA）收集非刺激性产生的泪液。所收集的泪液从角膜下缘收集，不能刺激到角膜、结膜或睑缘。收集 20μl样本后转移到0.5ml 的微管中，储存于-80 °C备用。

#### 7.4.2 炎症介质检测

根据制造商说明用Quantibody Human Inflammation Array I kit (RayBiotech, Inc. Norcross, GA)检测所收集的泪液中细胞白介素- 1α（interleukin-1α ，IL-1α），肿瘤坏死因子-α（tumor necrosis factor-α，TNF-α），神经生长因子（nerve growth factor ，NGF），干扰素-γ（interferon-γ ，IFN-γ），转化生长因子-β1（transforming

growth factor-β1，TGF-β1）和基质金属蛋白酶-9（matrix metalloproteinase-9 ，MMP-9）的表达。一抗孵育2小时后，生物素耦连的二抗孵育1小时。再与Cy3 标记过氧化酶孵育1小时，用GenePix 4000B (Bio-Rad Laboratories, Hercules, CA)检测表达，Quantibody® Q-Analyzer软件 (RayBiotech, Inc. Norcross, GA)分析结果。浓度根据制造商提供的标准曲线生成量化标准。

**8. 不良情况报告**

直接向钟兴武教授报告医疗安全问题。

电话：+86(757)68628481

**9. 终止或更改实验项目**

**9. 1** 研究对象可随时退出研究，同时，赞助人或研究者再认为研究对象不符合研究项目时也可终止对研究对象的观察。所有研究对象随时可因任何理由退出研究观察。项目支持研究者在研究中尝试合理的新方案，会详细记录在案。

**9.2** 研究对象中途退出研究后，结果不计入实验研究。

**10. 违反协议**

当研究对象未能遵守研究协议要求，影响纳入、排除标准、研究内容及目标时则按违反研究协议记录。违反协议内容包括：

1、不满足纳入或满足排除标准；

2、使用违禁药品。

**11. 研究管理，涉及伦理及监管**

此项研究将依据《赫尔辛基真言》采取保密措施。所有实验室标本、评分表格、实验报告及记录等均采用编码或首字母。资料将保存在文件柜中上锁保存，同时将研究对象的姓名与身份证件号码分开保存。未取得研究对象许可前，研究内容不会对外发布。研究人员也必需遵守相关隐私的法律法规。

### 14.1 伦理委员会

研究相关的协议书将有当地单位的伦理委员会审查、批准。严重违反伦理委员会的规定政策将会受到研究制约，同时也需向伦理委员会上报研究进度。研究人员需遵守伦理委员会的相关规定。变更研究计划执行前需重新递交书面申请，并通过伦理委员会审查，除变更后勤、管理方面以外。伦理委员会将更新研究项目申请。出现严重意外情况或不良反应时应按照伦理委员会的相关政策处理，收集研究不良影响的新信息，直到研究完成前，每年度需重新申请、审核研究项目。

### 14.2 计划修正

变更研究计划执行前需重新递交书面申请，并通过伦理委员会审查，除非必需立即消除病人的安全隐患。

### 14.3 知情同意书

按照《赫尔辛基宣言》和地方相关法律法规途经取得研究对象的知情同意书。知情同意书必需由研究资助人和伦理委员会双方确认、批准。研究者需向研究资助人提供伦理委员会审核的知情同意书副本。纳入研究对象时应先签署知情同意书。并向研究对象详细说明研究内容并征得同意。
